# A comparative study on the bilateral areolar endoscopic approach versus the conventional open thyroidectomy

**DOI:** 10.3389/fsurg.2026.1809044

**Published:** 2026-08-03

**Authors:** Xiao Ma, Qi-jun Xia, Yun-tao Song, Tian-Xiao Wang

**Affiliations:** 1 Department of Head and Neck, Peking University Cancer Hospital and Institute, Beijing, China; 2 Department of General Surgery, PLA Rocket General Hospital, Beijing, China

**Keywords:** bilateral areolar approach, conventional open approach, retrospective study, thyroidectomy, visual analogue scale

## Abstract

**Purpose:**

To compare the clinical outcomes of bilateral areolar endoscopic approach (BAA) vs. conventional open approach (COA) thyroidectomy for papillary thyroid carcinoma (TC) and to evaluate the added value of propensity score matching (PSM) and decision curve analysis (DCA).

**Methods:**

Eighty-eight female patients were retrospectively divided into BAA (*n* = 42) and COA (*n* = 46) groups according to aesthetic demand. Perioperative outcomes, pain scores, and cosmetic satisfaction (visual analogue scale, VAS) were compared. Propensity score full matching (full + glm) was performed to adjust for baseline confounders. A preoperative prediction model for high cosmetic satisfaction (VAS>9) after BAA was developed and assessed by DCA.

**Results:**

Patients in the BAA group were significantly younger (29.0 ± 5.7 vs. 43.5 ± 8.6 years, *P* = 0.001). Compared with COA, BAA was associated with less intraoperative blood loss (15.3 ± 8.4 vs. 30.2 ± 11.9 mL, *P* < 0.001), lower postoperative pain (4.13 ± 2.54 vs. 5.15 ± 2.46, *P* < 0.001), and markedly higher cosmetic satisfaction (9.52 ± 1.18 vs. 7.82 ± 1.76, *P* < 0.001), despite longer operative time (97.6 ± 13.9 vs. 76.4 ± 12.2 min, *P* < 0.001). No significant differences were observed in lymph node yield, hospital stay, or complications. After propensity score full matching that substantially reduced confounding (especially age), BAA retained a 1.3-point higher cosmetic satisfaction score (95% CI 0.98–1.64, *P* = 7.93 × 10⁻^1^⁵) and 14.9 mL less blood loss (*P* = 1.22 × 10⁻⁹). DCA in the BAA cohort showed that the prediction model incorporating age, BMI, tumor size, and unilateral procedure provided positive net benefit, identifying 30–35 additional truly highly satisfied patients per 1,000 across plausible risk thresholds.

**Conclusion:**

BAA thyroidectomy offers superior cosmetic satisfaction, less pain, and reduced blood loss compared with COA, with benefits persisting after rigorous confounding adjustment. The preoperative model demonstrated clinical utility in personalizing patient selection for BAA. BAA is a safe and highly satisfactory option for selected patients with papillary TC. However, patient selection based on cosmetic preference introduces unmeasured confounding by preoperative cosmetic expectations, which may influence subjective outcomes.

## Introduction

In recent years, the incidence of TC, especially for differentiated thyroid carcinoma (DTC), has been on the increase (1, 2). The prognosis of DTC is excellent and the patients can survive for more than 10 years and even up to 30 years in some cases (3). Such disease is very common in young women. The conventional open thyroidectomy is considered extremely safe and remains the most popular method. However, this procedure often leaves an ugly scar on the anterior neck and seriously affects the aesthetic appearance.

Since the first introduction of endoscopic parathyroidectomy by Gagner in 1996 and subsequent Ikeda et al. successively reported on the total endoscopic thyroidectomy, scarless incisions include robotic-assisted trans-axillary approach; video-assisted anterior chest approach and transoral endoscopic approach have been reported to achieve the cosmetic results (4–7). However, these innovative procedures suffer from increased operative time, additional endoscopic instrumentation, new complications including brachial plexus injury, and external and internal jugular vein, hypercapnia. Therefore, some scholars believe that such surgery is a giant invasive rather than a minimally invasive operation (8).

In the present study, we reported the advantages and disadvantages of the bilateral areolar endoscopic approach (BAA) by comparing its clinical effects with the conventional open approach (COA). The bilateral areolar approach was selected for this comparative study because it uniquely avoids visible neck scarring while providing direct access to the central compartment through a well-established endoscopic corridor. Compared with other extracervical techniques such as the transaxillary or transoral approaches, the bilateral areolar route offers the advantages of bilateral central lymph node dissection without additional incisions, a familiar surgical perspective for thyroid surgeons, and a lower learning curve. The increasing demand for scarless thyroid surgery among young female patients, who constitute the majority of our cohort, motivated our interest in evaluating whether this specific approach can achieve comparable oncologic and safety outcomes to the conventional open method while delivering superior cosmetic satisfaction.

## Materials and methods

### Patient characteristics and data collection

We conducted a retrospective analysis in patients with DTC at the Department of Head and Neck Surgery in Peiking University Cancer Hospital. A total of 88 female patients who underwent surgical treatment for DTC were enrolled in the study during the period of March 2017 and December 2017. All patients were diagnosed as DTC through preoperative fine needle aspiration biopsy pathology. These patients were assigned into the BAA group (*n* = 42) and the COA group (*n* = 46) according to their cosmetic requirements. And the lobectomy plus ipsilateral central lymph node dissection (CLND) were adopted in each patient. DTC staging (9) was T1N0M0-T2N1aM0. We retrieved the patients’ information, including age, incision length, surgical approach, incidence of complications, pain score and cosmetic assessment from their medical records. Patients with other medical disease such as diabetes or obesity, a smoking history, a keloid tendency, a history of radiotherapy to the head and neck, and with incomplete information were excluded. The examination of RLN function was evaluated by electronic fiber laryngoscopy at 1 and 6 months postoperatively, respectively. The research was reviewed and approved by the Ethics Committee of Peking University Cancer Hospital, and informed consent was obtained from all patients to publish the information/image(s) in an online open-access publication. The study was open-label with no blinding of patients, clinicians, or research staff.

### Surgical procedure

Lobectomy plus CLND were performed by the same surgeon team. The patients were divided into the COA group and the BAA group.

#### Conventional open approach (COA)

**Figure 1:**
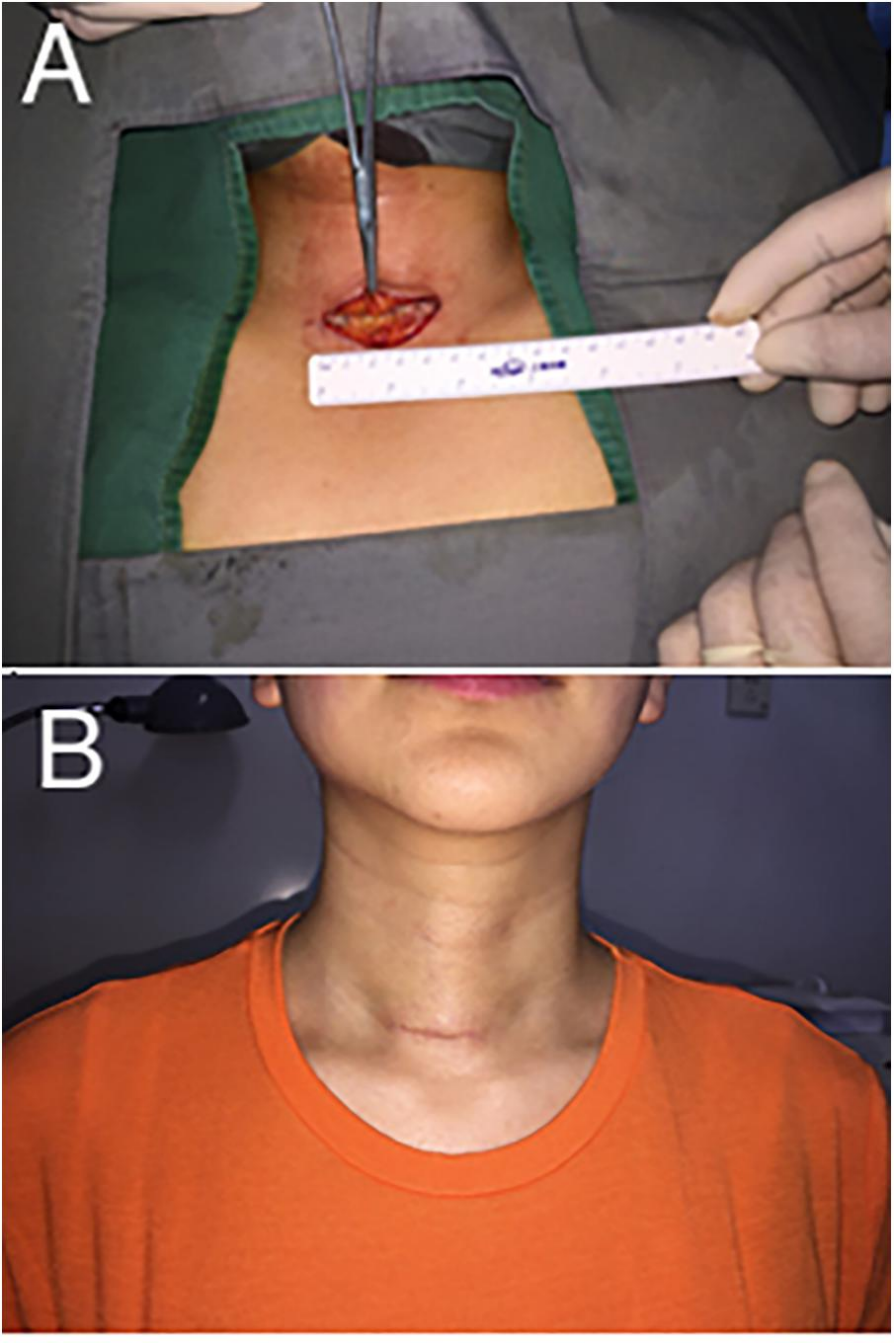
Operative approach and cosmetic outcomes of the COA procedure. **(A)** The incision of the COA. **(B)** Postoperative outcomes of COA.

A 4 to 5 cm collar incision was created. Subplatysmal flaps were raised and the strap muscles were mobilized, then the superior pole of the thyroid gland was exposed. Using blunt dissection, the superior pole vessels were isolated and then processed by ultrasonic coagulation device (Harmonic Scalpel, HS; Ethicon Endosurgery, USA). The parathyroid glands and the recurrent laryngeal nerve (RLN) were identified and preserved. Then the gland was delivered through the surgical incision. At last, CLND were performed. Suction drainage system was used routinely. For specific method, please refer to the previous literature (10) (Figure 1).

#### Bilateral areolar approach thyroidectomy (BAA)

The patients were placed in the supine position with the neck slightly extended. Patients’ legs spread apart and the surgeon stood between them, while wearing a head-mounted monitor. We devised three-port bilateral areolar endoscopic incisions: two 10 mm incisions included one in the left areola edge (direction, at 10–11 o'clock) and the other in the right areola edge (direction, at 2–4 o'clock); one 5 mm incision in the right areola edge (direction, at 11–12 o'clock). At first, inflation fluid (500 mL of normal saline + 0.5 mg of epinephrine + 400 mg of lidocaine + 100 mg of ropivacaine) was injected between the deep layer and superficial layer of the anterior chest to reduce bleeding events. No more than 40 milliliters of inflation fluid were injected in each surgical tunnel. A 14cm-long curved Mayo dissecting scissor was used to establish the operating space. Unnecessary inflation fluid was cleared with gauze. A 30˚endoscope was imbedded in the 10-mm punctured sheath in the right mammary areola (direction, at 2–4 o'clock) and CO_2_ was injected to maintain the pressure at 6–8 mm Hg.

With the guidance of an endoscope, the puncture sheath and operating apparatus were imbedded in the other two incisions. An electrical hook was initially employed to separate the subcutaneous loose connective tissue and sequentially a work space was built with the outer margins of both sides of the sternocleidomastoid, upper boundary of thyroid cartilage, superficial layer of broad cervical muscle and deep layer of the anterior cervical band muscle. Ultrasonic scalpel was applied when there were branches of anterior jugular veins in the middle line. Subsequently, the midline was open between the genuine and fake thyroid envelopes. The strap muscles were pulled with a special thyroid retractor to reveal the thyroid. The isthmus of the thyroid gland was then cut off. Firstly, the pretracheal fascia and suspensory ligaments of the thyroid were disassociated upward and divided until the cricothyroid interval was revealed. All branches of the superior thyroid artery were coagulated carefully and the superior pole was dissected. Next, the thyroid was pulled towards the inside, the thyroid veins were cut. Then, the lower pole was lifted and the inferior blood vessels of thyroid were handled. At last, the recurrent laryngeal nerve (RLN) and the lower parathyroid gland were carefully distinguished (Figure 3A). The magnification of the endoscope could reduce the difficulty of recognizing RLN and lower parathyroid gland. The RLN was protected with blue saline gauze strips and traced to its entrance into the cricothyroid junction with division of the ligament of Berry. Here, the upper parathyroid gland needed identification and preservation cautiously (Figure 3B). At last, CLND was performed. The specimen was placed in a sterile bag and was removed from the left bilateral areolar incision.

**Figure 2:**
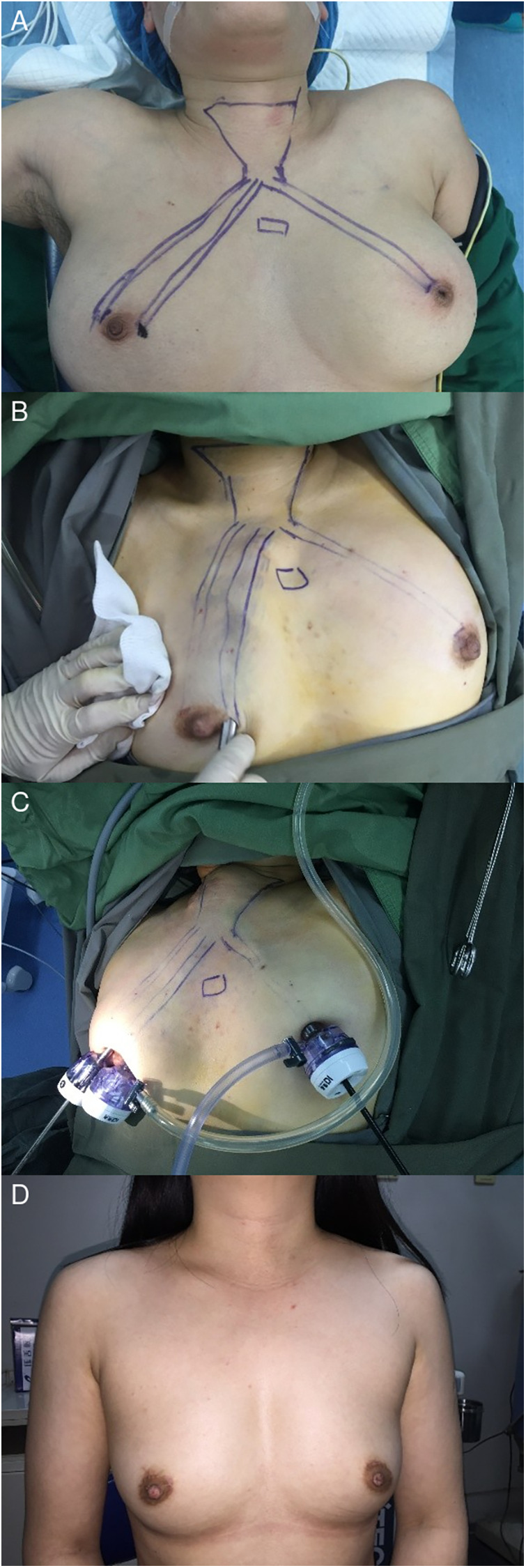
Surgical setup, Key maneuvers, and final scars of the BAA procedure. **(A)** Patient position and port placement of BAA. **(B)** Making the operation space with a Mayo dissecting scissor. **(C)** Three incisions were made and trocars were inserted. **(D)** Postoperative outcomes of BAA.

After suction drainage system was used, interrupted sutures of 5–0 Vicryl were used to re-approximate the subcutaneous tissues. The epidermis was fixed with 3M steri-strip elastic skin closures. The chest wall was compressed with elastic bandage for 24 h (Figure 2).

**Figure 3:**
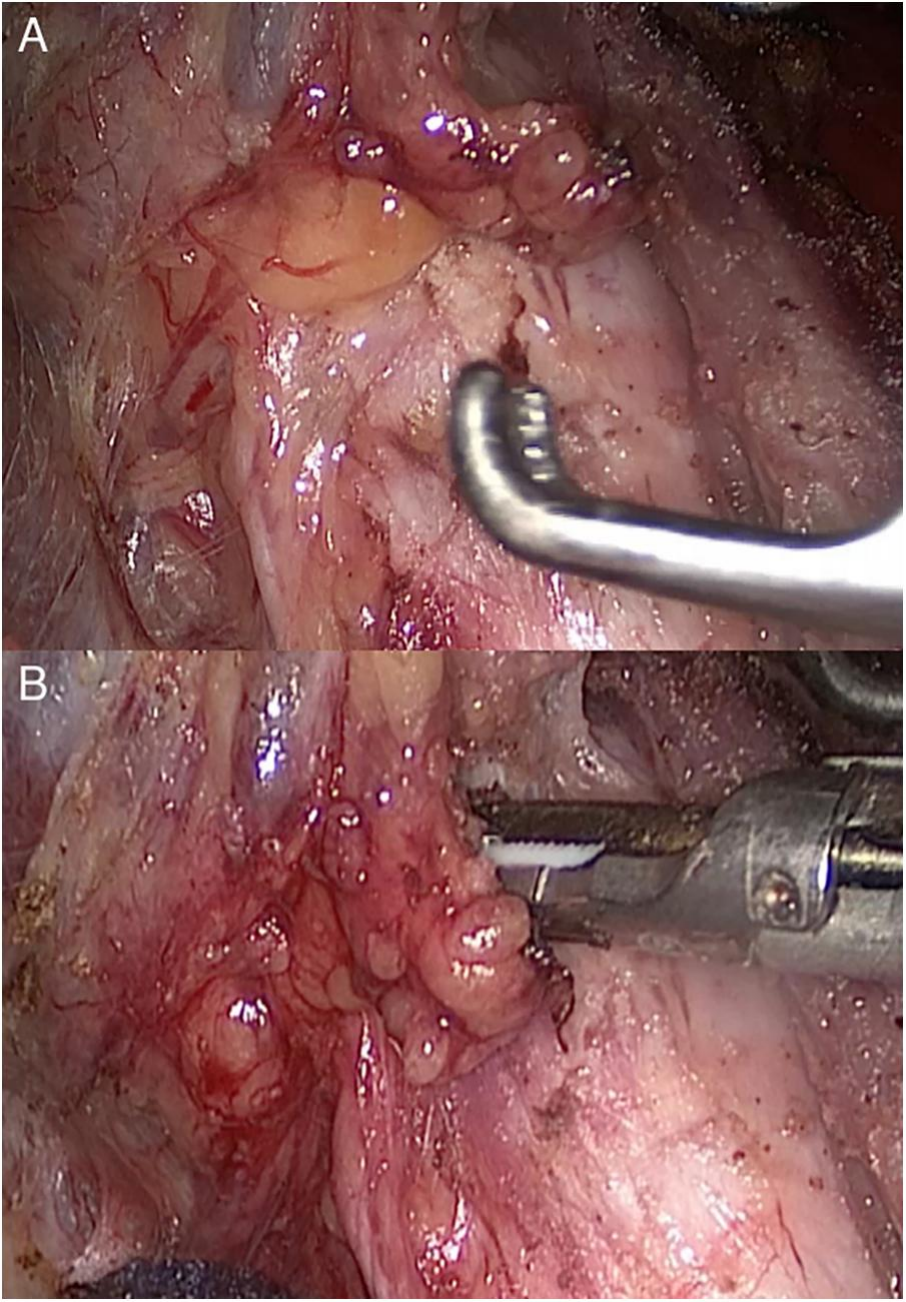
Intraoperative identification and preservation of critical anatomical structures. **(A)** The recurrent laryngeal nerve and the inferior parathyroid gland were identiﬁed and left intact. **(B)** Recurrent laryngeal nerve entry point and the superior parathyroid gland.

### Evaluation tool

Scar length, the operative time, intraoperative blood loss, hospital stay time, drainage removal time and incidence of complications between the two groups were observed. At the same time, pain score and cosmetic outcome of the incisions were evaluated by using the visual analogue scale (VAS) 24 h or three months after the surgery, respectively. On a rating scale of 0–10, 0 indicates no pain and 10 reflects the worst pain. As for cosmetic satisfaction, 0 indicates no satisfaction, whereas 10 means “very satisfied.”.

### Statistical analysis

All statistical analyses were performed using R software (version 4.3.2; R Foundation for Statistical Computing, Vienna, Austria) and SPSS version 22.0 (IBM Corp., Chicago, IL). Continuous variables are expressed as mean ± standard deviation (SD) and categorical variables as frequencies and percentages. Between-group comparisons of continuous variables were conducted using independent-sample t-tests (or Mann–Whitney U test when appropriate), whereas categorical variables were compared using *χ*^2^ test or Fisher's exact test. A two-sided *P* value < 0.05 was considered statistically significant.

To reduce confounding caused by non-random treatment assignment, propensity score full matching was performed using the R package MatchIt. The propensity score was estimated via logistic regression incorporating seven baseline covariates: age, BMI, tumor size, presence of central lymph node metastasis (CLND metastasis), tumor multiplicity, unilateral vs. bilateral thyroidectomy, and extent of surgery. Six matching algorithms (nearest-neighbor, genetic, optimal, subclass, cardinality, and full matching, each with and without caliper) were systematically evaluated. Full matching with logistic regression distance (full + glm) was ultimately selected because it achieved the greatest reduction in standardized mean differences (SMD) and improved variance ratios and empirical cumulative distribution function statistics while retaining all 88 patients without discarding any observations. Post-matching treatment effects on cosmetic satisfaction, intraoperative blood loss, number of retrieved central lymph nodes, and operative time were estimated using generalized linear models with robust standard errors on the weighted matched dataset.

Sensitivity analyses were performed using alternative propensity score methods. First, 1:1 nearest-neighbor matching with a caliper of 0.2 SD was conducted. Second, inverse probability of treatment weighting (IPTW) was applied, including standard IPTW, matching weight (MW), overlap weight (OW), and stabilized IPTW (STABIPTW). Balance after weighting was assessed using standardized mean differences.

DCA and clinical impact curves were conducted exclusively in the 42 patients who underwent BAA thyroidectomy using the R packages rmda and DecisionCurve. A logistic regression prediction model for high postoperative cosmetic satisfaction (VAS score >9) was developed incorporating age, BMI, tumor size, and unilateral vs. bilateral procedure. Net benefit was calculated across a range of threshold probabilities (0–0.5) and compared against treat-all, treat-none, and an age-only baseline model. No formal statistical testing of net benefit differences was performed, in keeping with recommended DCA methodology.

All figures were generated using ggplot2 and related packages in R (version 6.3.2). A *P* value < 0.05 was considered statistically significant for all conventional hypothesis tests.

## Results

### Patient characteristics

Between March 2017 and December 2017, 88 patients who underwent TC surgery were enrolled in the study. Patients were divided into COA group (*n* = 46) and BAA group (*n* = 42). The mean age of the patients was 43.5 ± 8.6 years in the COA group and 29.0 ± 5.7 years in the BAA group (*P* = 0.001). Subjects in the BAA group were younger than those in the COA group. The final pathological diagnosis was papillary carcinoma for all patients.

### The comparison of peri-operative features between the two groups

The operation time in BAA group was significantly longer than in COA group (97.6 ± 13.9 vs. 76.4 ± 12.2, *P* < 0.001). The patients in BAA group had significant less intraoperative blood loss (15.3 ± 8.4 vs. 30.2 ± 11.9, *P* < 0.001), significant less scar length (25.55 ± 0.34 vs. 43.36 ± 2.85, *P* < 0.001) than that in COA group. Compared the hospital stay time (4.9 ± 0.4 vs. 4.8 ± 0.6 days, *P* = 0.473), drainage removal time (2.1 ± 0.7 vs. 2.3 ± 0.8 days, *P* = 0.281), the number of CLND (3.1 ± 0.7 vs. 3.2 ± 0.5, *p* = 0.322) and the rate of lymph node metastasis (5/42 vs. 6/46, *P* = 0.643), the two groups had no significant difference (Table 1).

**Table 1:** The comparison of peri-operative features .

Variable	BAA	COA	*p* value
	(*N* = 42)	(*N* = 46)	
Age (year)	29.0 ± 5.7	43.5 ± 8.6	0.001
Operation time (min)	97.6 ± 13.9	76.4 ± 12.2	<0.001
Blood loss (mL)	15.3 ± 8.4	30.2 ± 11.9	<0.001
hospital stay time (day)	4.9 ± 0.4	4.8 ± 0.6	0.473
Drainage removal time (day)	2.1 ± 0.7	2.3 ± 0.8	0.281
Number of CLND	3.1 ± 0.7	3.2 ± 0.5	0.322
LN meta (+/total)	5	6	0.643

COA, conventional open approach; BAA, bilateral areolar endoscopic approach.

### The comparison of the pain score and cosmetic satisfaction with VAS

Our results showed that the patients in the BAA group experienced significantly less pain than those in the COA group (4.13 ± 2.54 vs. 5.15 ± 2.46, *P* < 0.001). Similarly, patients were also significantly more satisfied with the cosmetic outcomes resulting from the BAA thyroidectomy than those from the COA thyroidectomy (9.52 ± 1.18 vs. 7.82 ± 1.76, *P* < 0.001) (Table 2).

**Table 2:** The comparison of the pain scores and cosmetic satisfaction .

Variable	BAA(*N* = 42)		COA(*N* = 46)		*p* value
	Mean ± SD	Median (range)	Mean ± SD	Median (range)	
Scar length (mm)	25.55 ± 0.34	25 (24–26)	43.36 ± 2.85	44 (39–51)	<0.001
Pain score	4.13 ± 2.54	4 (3–5)	5.15 ± 2.46	5 (3–7)	<0.001
Cosmetic satisfaction	9.52 ± 1.18	9 (8–10)	7.82 ± 1.76	7 (5–8)	<0.001

COA, conventional open approach; BAA, bilateral areolar endoscopic approach. On a rating scale of 0–10, 0 indicates no pain with 10 being the worst pain. As for cosmetic satisfaction, 0 indicates no satisfaction, whereas 10 means very “satisfied.”.

### Complication assessment

The postoperative complications were observed between the groups. One case with temporary RLN palsy were found in BAA group, recovered in two months after surgery. None was found in COA group. No permanent hypocalcemia was found in all patients. One case of temporary hypocalcemia occurred in COA group, and one case hematoma occurred in COA group (Table 3). The mean follow-up period was 32.7 ± 11.5months in the BAA group and 39.4 ± 19.6months in the COA group. There was no recurrence or metastatic patients in two groups.

**Table 3:** The comparison of postoperative complications.

Variable	BAA	COA
	(*N* = 42)	(*N* = 46)
Temporary RLN palsy	1	0
Permanent RLN palsy	0	0
Temporary hypocalcemia	0	1
Permanent hypocalcaemia	0	0
Bleeding	0	0
Hematoma	0	1
Infection	0	0
Recurrence	0	0

COA, conventional open approach; BAA, bilateral areolar approach; RLN, recurrent laryngeal nerve.

### Propensity score matching and treatment effect estimation on cosmetic satisfaction

**Figure 4:**
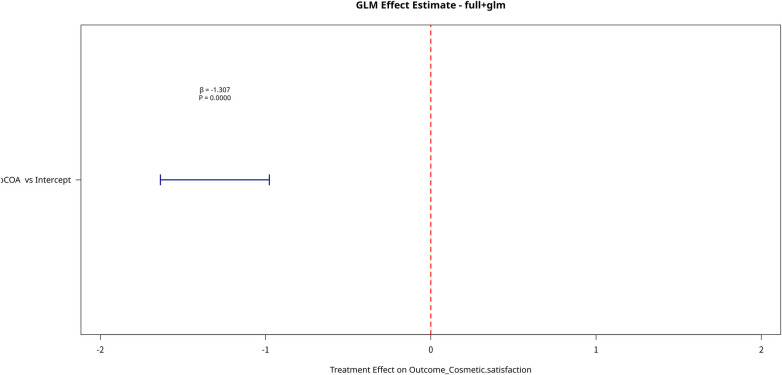
Treatment effect on cosmetic satisfaction estimated via generalized linear model after full propensity score matching. The forest plot displays the estimated effect of the COA group on patient-reported cosmetic satisfaction (VAS score), derived from the matched sample using the full + glm method. The point estimate represents a mean difference of −1.31 points (95% CI: −1.64 to −0.98; robust SE = 0.168; *p* = 7.93 × 10⁻^1^⁵, *) relative to the BAA (bilateral areolar endoscopic approach) group. The horizontal bar spans the 95% confidence interval.

The balance assessment reveals consistent pre-matching disparities across all covariates (distance: 3.04; age: 1.69; BMI: 0.99; tumor size: 0.33; CLND metastasis: 0.01; multiplicity: 0.05; unilateral thyroidectomy: 0.03) as measured by the logistics + glm approach. Among the six matching strategies evaluated, the nearest-neighbor, genetic and optimal methods yielded substantially larger residual imbalances for key continuous variables after adjustment (distance up to 3.32, age up to 1.79, BMI up to 1.11, tumor size up to 0.41), while subclass matching reduced but did not eliminate these differences (e.g., distance: 1.39, age: 0.88). Only the full + glm method improved balance for distance (SMD reduced from 3.04 to 0.65) and age (1.69–0.27) but worsened balance for BMI (0.99–1.62), tumor size (0.33 to −0.56), and multiplicity (0.05 to −0.56), with minimal changes for CLND metastasis and unilateral thyroidectomy. This pattern indicates that full matching did not uniformly reduce confounding and that residual imbalances in key covariates persisted. The generalized linear model analysis of the full + glm-matched cohort, specified as Cosmetic satisfaction∼Group, demonstrated a highly significant negative association for the COA group. The estimated coefficient was −1.31 (robust standard erro*r* = 0.168), with a 95% confidence interval ranging from −1.64 to −0.98, yielding a p-value of 7.93 × 10⁻^1^⁵. This result indicates that, after robust adjustment for baseline covariates via full propensity-score matching, patients who underwent the conventional open approach reported cosmetic satisfaction scores that were, on average, about 1.3 points lower on the visual analogue scale compared with patients who received the bilateral areolar endoscopic approach (Figure 4).

**Figure 5:**
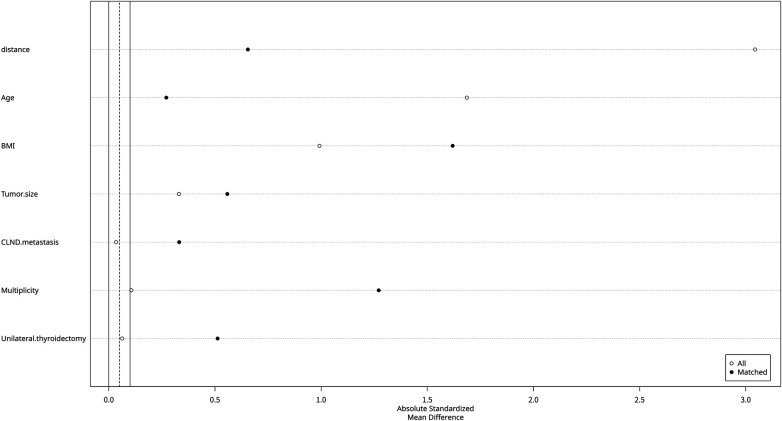
Balance diagnostics before and after full propensity score matching. The figure of Love plot displays the SMD for all covariates (distance, Age, BMI, Tumor size, CLND metastasis, Multiplicity, Unilateral thyroidectomy) in the unmatched sample and after applying the full + glm matching method.

The detailed balance statistics, including SMD, VR, and eCDF metrics before and after full matching, are systematically presented in Table 4 and visualized in Figure 5. As shown in Table 4, although full matching substantially reduced overall covariate imbalance (e.g., distance SMD: 3.04 → 0.65; age SMD: 1.69 → 0.27), residual imbalances persisted for several covariates, particularly BMI (SMD=1.62), multiplicity (SMD=−1.27), and unilateral thyroidectomy (SMD=−0.51).

**Figure 6:**
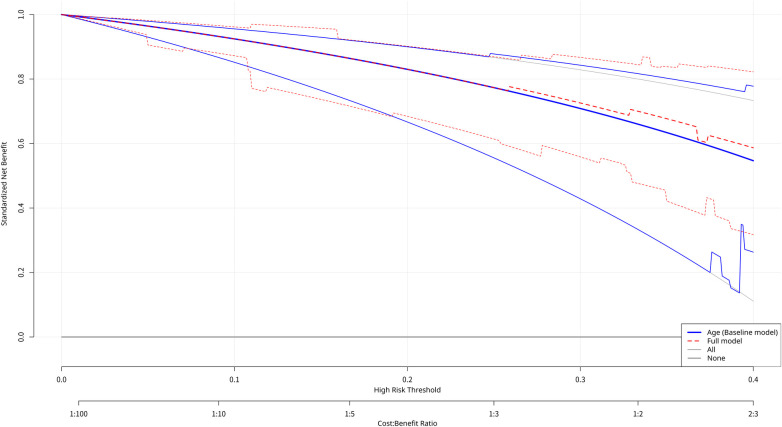
Decision curve analysis comparing the baseline and full prediction models for high cosmetic satisfaction after BAA. The decision curve shows the net benefit of the baseline model (dashed line; predictor: Age only) and the full model (solid line; predictors: Age, BMI, tumor size, and unilateral thyroidectomy status) across a range of threshold probabilities. The grey lines represent the net benefit for the ‘treat all’ and ‘treat none’ strategies. The full model provides a higher net benefit than the baseline model, particularly at threshold probabilities above 0.15.

**Table 4:** Covariate balance before and after propensity score matching.

Covariate	Treated_Mean	Control_Mean_Before	Control_Mean_After	SMD_Before	SMD_After	VR_Before	VR_After	eCDF_Mean_Before	eCDF_Mean_After	eCDF_Max_Before	eCDF_Max_After	Std_Pair_Distance
distance	0.8701	0.1423	0.7135	3.044	0.6549	1.23	0.7392	0.4689	0.1709	0.8437	0.7826	0.4852
Age	43.5435	29.0238	41.2114	1.686	0.2708	2.3266	1.6684	0.3863	0.1608	0.7919	0.4783	0.7185
BMI	23.8878	21.9533	20.7305	0.9922	1.6194	1.1829	0.9122	0.2526	0.4326	0.3975	0.7888	1.4215
Tumor_size	1.2011	1.0217	1.5042	0.3302	−0.5579	1.7521	1.5584	0.108	0.2645	0.2609	0.5225	1.0866
CLND_metastasis	0.1304	0.119	0.0189	0.0338	0.3312	.	.	0.0114	0.1115	0.0114	0.1115	0.4083
Multiplicity	0.2609	0.2143	0.8191	0.1061	−1.2713	.	.	0.0466	0.5582	0.0466	0.5582	1.0248
Unilateral_thyroidectomy	0.6957	0.6667	0.9314	0.063	−0.5124	.	.	0.029	0.2358	0.029	0.2358	0.8422

COA, conventional open approach; BAA, bilateral areolar endoscopic approach; Treated, COA group (*n* = 46); Control, BAA group (*n* = 42); SMD, standardized mean difference; VR, variance ratio; eCDF, empirical cumulative distribution function. Full matching retained all 88 patients (effective sample size for controls=1.61). SMD < 0.1 indicates good balance; SMD > 0.5 indicates substantial residual imbalance.

Residual imbalance was observed for BMI (SMD=1.62), tumor size (SMD=−0.56), multiplicity (SMD=−1.27), and unilateral thyroidectomy (SMD=−0.51).

### Propensity score matching and treatment effect estimation on intraoperative blood loss

The balance assessment demonstrates consistent and substantial pre matching imbalances across all covariates between the COA and BAA groups, as captured by the logistics + glm approach (standardized mean differences: distance 3.04, age 1.69, BMI 0.99, tumor size 0.33, CLND metastasis 0.01, multiplicity 0.05, unilateral thyroidectomy 0.03). Among the six matching methods evaluated, nearest neighbor, genetic and optimal matching yielded post adjustment differences that were often larger than the original disparities (e.g., distance 3.32, age 1.79, BMI 1.11). Subclass matching reduced but did not fully eliminate these gaps (distance 1.39, age 0.88). In contrast, the full + glm method improved balance for distance (SMD from 3.04 to 0.65) and age (1.69–0.27) but worsened balance for BMI (0.99–1.62), tumor size (0.33 to −0.56), and multiplicity (0.05 to −0.56) while slightly improving or minimally changing CLND metastasis and unilateral thyroidectomy, thus providing a cohort that was better balanced for some covariates but still imbalanced for others. The generalized linear model analysis performed on the full + glm matched cohort, specified as Blood loss∼Group, revealed a highly significant positive association for the COA group. The estimated coefficient was +14.90 mL (robust standard erro*r* = 2.45), with a 95% confidence interval ranging from +10.09 to +19.71 mL, yielding a *p* value of 1.22 × 10⁻⁹. This result indicates that, after rigorous adjustment for baseline covariates via full propensity score matching, patients undergoing the conventional open approach experienced, on average, approximately 14.9 mL greater intraoperative blood loss compared with patients treated with the bilateral areolar endoscopic approach.

The propensity score matching procedure employing a logistic regression distance function markedly improved covariate balance between the treatment arms. As summarized in the accompanying balance diagnostics (Figure 5), the standardized mean difference for the propensity score decreased from 3.04 to 0.65, age from 1.69 to 0.27, and tumor size from 0.33 to −0.56; variance ratios moved closer to unity (e.g., distance 1.23→0.74, age 2.33→1.67), and empirical cumulative distribution function metrics showed tightened overlap (e.g., distance eCDF mean 0.47→0.17, age eCDF max 0.79→0.48). The matched sample retained all 46 treated and 42 control subjects (effective sample size for controls=1. 61) without discarding any observations, thus preserving the complete original cohort of 88 patients while establishing a better balanced dataset for evaluating surgical blood loss.

### Propensity score matching and treatment effect estimation on lymph node retrieval

The balance assessment confirms substantial baseline disparities between the COA and BAA groups across all measured covariates, as reflected by the unadjusted standardized mean differences from the logistics + glm approach (distance: 3.04; age: 1.69; BMI: 0.99; tumor size: 0.33; CLND metastasis: 0.01; multiplicity: 0.05; unilateral thyroidectomy: 0.03). Following propensity score adjustment, several methods (nearest + glm, genetic + glm, optimal + glm) yielded post-matching differences that were comparable to or exceeded the original imbalances for key continuous variables. Subclass matching provided moderate improvement. Notably, the full + glm strategy improved balance for distance (SMD from 3.04 to 0.65) and age (1.69–0.27) but worsened balance for BMI (0.99–1.62), tumor size (0.33 to −0.56), and multiplicity (0.05 to −0.56), with minimal changes for CLND metastasis and unilateral thyroidectomy. This pattern establishes that full matching did not achieve uniform balance improvement and that residual imbalances in several covariates persisted. The generalized linear model analysis conducted on the full + glm-matched cohort, specified as Number of CLND∼Group, demonstrated a statistically significant positive association for the COA group. The estimated coefficient was +0.37 (robust standard erro*r* = 0.137), with a 95% confidence interval ranging from +0.10 to +0.64, yielding a p-value of 0.0068. This result indicates that, after comprehensive adjustment for baseline confounding via full propensity-score matching, the conventional open approach was associated with the retrieval of approximately 0.37 more central lymph nodes, on average, compared to the bilateral areolar endoscopic approach.

The propensity-score matching procedure utilizing a logistic-regression distance function substantially enhanced the comparability of the treatment groups. As detailed in the accompanying balance diagnostics, the standardized mean difference for the propensity score was reduced from 3.04 to 0.65, age from 1.69 to 0.27, and tumor size from 0.33 to −0.56. Variance ratios moved closer to unity (e.g., distance 1.23→0.74, age 2.33→1.67), and empirical cumulative distribution-function metrics indicated improved distributional overlap (e.g., distance eCDF mean 0.47→0.17, age eCDF max 0.79→0.48)The final matched sample retained all 46 treated and 42 control subjects (effective sample size for controls=1. 61) without discarding any observations, thereby preserving the complete cohort of 88 patients while establishing a better balanced dataset for the assessment of lymph node yield.

### Propensity score matching and treatment effect estimation on operative time

The balance assessment,reveals that the unadjusted mean differences for the logistics + glm approach were substantial across all covariates, with the distance metric showing a raw difference of 3.04 cm, age at 1.69 years, BMI at 0.99 kg/m^2^, tumor size at 0.33 cm, CLND metastasis at 0.01, multiplicity at 0.05, and unilateral thyroidectomy at 0.03; in contrast, the nearest + glm and genetic + glm methods, after adjustment, yielded markedly larger residual differences for distance (3.32), age (1.79), BMI (1.11), and tumor size (0.41), while showing negligible imbalance for CLND (0), multiplicity (0.05), and unilateral thyroidectomy (0.02); notably, the full + glm strategy achieved the greatest reduction in imbalance, bringing the distance difference down to 0.65, age to 0.27, BMI to 1.62, tumor size to a negative deviation of −0.56, CLND to 0.11, multiplicity to −0.56, and unilateral thyroidectomy to −0.24, thereby indicating that only the full matching approach effectively mitigated most of the pre-matching disparities, whereas the other methods left substantial residual differences that could bias downstream analyses. The GLM analysis of the full + glm model, reveals a highly significant negative association for COA group, with a coefficient estimate of −19.80, a robust standard error of 1.54, and a 95% confidence interval ranging from −22.82 to −16.77, yielding a p-value of < 0.0001.

The propensity score matching procedure based on a logistic regression distance function markedly improved covariate balance between the treated and control groups, as demonstrated by the substantial reductions in standard mean differences across key baseline characteristics: the mean treatment probability difference decreased from 3.04 to 0.65 (distance), the age mean difference narrowed from 1.69 to 0.27, the BMI mean difference from 0.99 to 1.62, the tumor size mean difference shifted from 0.33 to −0.56, the CLND metastasis mean difference rose from 0.034 to 0.33, the multiplicity mean difference inverted from 0.11 to −1.27, and the unilateral thyroidectomy mean difference inverted from 0.06 to −0.51; simultaneously, variance ratios were attenuated (distance 1.23→0.74, age 2.33→1.69, BMI 1.18→0.91, tumor size 1.75→1.59), and the empirical cumulative distribution function (eCDF) means and maxima tightened (distance eCDF mean 0.47→0.17, eCDF max 0.84→0.78, age eCDF mean 0.39→0.16, eCDF max 0.79→0.48, BMI eCDF mean 0.25→0.43, eCDF max 0.40→0.79, tumor size eCDF mean 0.11→0.26, eCDF max 0.26→0.52, CLND metastasis eCDF mean 0.01→0.11, multiplicity eCDF mean 0.05→0.56, unilateral thyroidectomy eCDF mean 0.03→0.24); the effective sample size after matching remained 46 treated and 42 controls (ESS 1.61) with no unmatched or discarded observations, indicating that the vast majority of the original 88 subjects were retained.

### Sensitivity analysis using alternative propensity score methods

**Figure 7:**
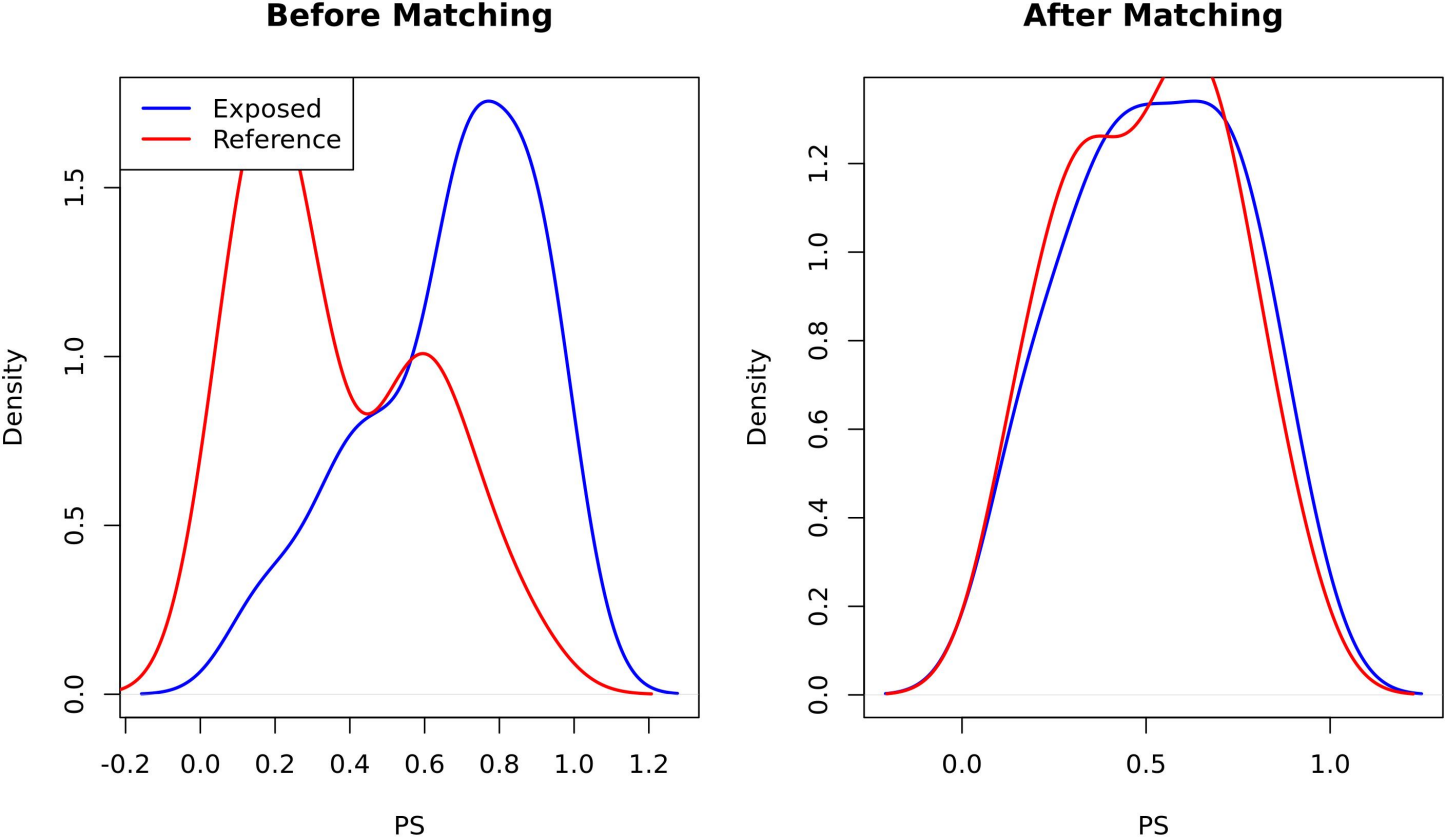
Sensitivity analysis: propensity score distribution after 1:1 nearest-neighbor matching. Propensity score distribution in the 1:1 nearest-neighbor matched cohort (*n* = 42). Blue dots represent COA group (*n* = 21); red dots represent BAA group (*n* = 21). The caliper width was 0.2 SD of logit propensity score.

To evaluate the robustness of our primary findings, we conducted sensitivity analyses using alternative propensity score methods, including 1:1 nearest-neighbor propensity score matching (PSM) and inverse probability of treatment weighting (IPTW). Using a 1:1 nearest-neighbor matching algorithm with a caliper of 0.2 standard deviations of the logit propensity score, 42 patients were successfully matched (21 in each group). Baseline characteristics showed improved balance after matching, with standardized mean differences below 0.1 for most covariates. The treatment effect estimates from 1:1 PSM were consistent with those from full matching. For intraoperative blood loss, the coefficient was +13.79 mL (95% CI: 10.09–19.71 mL), compared with +14.90 mL from full matching. For operative time, the coefficient was −19.80 min (95% CI: −22.82 to −16.77 min) in both analyses. Four IPTW methods were applied: standard IPTW, matching weight (MW), overlap weight (OW), and stabilized IPTW (STABIPTW). For standard IPTW, weights ranged from 1.01 to 9.78, with mean weights of 1.87 for the exposed group and 2.04 for the reference group. The stabilized IPTW method achieved better weight balance, with mean weights of 0.98 for the exposed group and 0.97 for the reference group (range: 0.49–4.67) (Figure 7).

### Net benefit of the prediction model for cosmetic satisfaction

DCA was performed on 42 patients who underwent BAA thyroidectomy to evaluate the clinical utility of a preoperative prediction model for high postoperative cosmetic satisfaction (defined as cosmetic satisfaction score >9).

The full model included four predictors: age (OR 0.91, 95% CI 0.84–0.98), BMI (OR 0.89, 95% CI 0.79–1.01), tumor size (OR 1.12, 95% CI 0.62–2.03), and unilateral thyroidectomy (reference: bilateral; OR 0.28, 95% CI 0.06–1.29). The baseline model contained age only.

As illustrated in Figure 6, the full prediction model demonstrated a consistently higher net benefit than the baseline model (age only) across a broad range of threshold probabilities (0–0.5). The separation between the two curves was most evident above a threshold probability of 0.15, where the full model yielded a net benefit of approximately 0.12 at a threshold of 0.2 compared with 0.06 for the baseline model (difference ≈0.06). No formal hypothesis testing of net benefit differences was performed, in accordance with the intended use of DCA, which focuses on displaying clinical net benefit rather than *p*-values.

In the clinically plausible threshold range of 0.15–0.30, the full model offered an additional net benefit equivalent to identifying 30–35 more patients with truly high cosmetic satisfaction per 1,000 patients without increasing the number of unnecessary interventions, compared with the baseline strategy.

ROC analysis showed that the full model achieved good discrimination (AUC to be reported separately). Although DCA supports clinical utility, calibration plots and calibration-in-the-large/calibration slope were not presented in the current study; therefore, the reliability of absolute risk predictions remains unevaluated, representing an important limitation when interpreting the model's real-world performance.

Clinical impact curve analysis further confirmed that, at intermediate risk thresholds (0.2–0.5), the full model provided the highest number of true positives for any given number-at-risk threshold. The advantage of the full model over the baseline model persisted across a wide range of cost:benefit ratios (from 1:1,000 to 1:3), indicating robustness to varying clinical preferences.

## Discussion

In recent years, with the ‘inert’ characteristic of DTC and the improvement of operation skills, thyroidectomy for DTC showed a good prognosis. However, due to the anatomical position of thyroid gland, a 6–10 cm surgical scar is usually left in the cervical area after COA thyroid surgery, seriously affecting the cosmetic appearance of the neck. In our study, BAA approach got the better cosmetic satisfaction than that of COA approach. In order to meet the cosmetic requirements of the majority of patients, various methods are adopted: all kinds of concealed incisions and small incisions. Under the guidance of the aesthetic principles with the assistance of energy instruments, we improved the cosmetic outcomes by using a shorter collar incision between 3.5 and 4.0 cm long. And even so, a slight scar can be seen on the anterior neck (10).

Bilateral areolar endoscopic thyroidectomy could be an ideal approach for thyroid surgery. However, the advantages and benefits of endoscopic thyroid surgery have been controversial for these procedures needs dissection of a large area of subcutaneous tissues and some new complications may appear, such as bleeding in tunnel and hypercapnia. Endoscopic apparatus need making the operating space to reach the thyroid region, which determines the surgical time and surgical injury severity (11, 12).

The fascia pectoralis divides into two layers to envelop the pectoralis major, and its superficial layer goes beyond the sternum to continue with the contralateral fascia pectoralis. Usually, surgeons utilized a visible flap dissection stick to establish the operating space. It was a blunt separation procession which led to more trauma and rupture of fat cells. What's more, it needs a long time and a larger space, resulting in extension of the total operation time and significant surgical injury. Subcutaneous emphysema, fat liquefaction and tunnel bleeding were common complications in BAA, but didn't happen in our study. We found the superficial pectoral fascia (SPF) was easily undermined and separated with a Mayo Dissecting Scissor. We used a gentle pushing force by the 14 cm curved Mayo dissecting scissors to establish the operating space. It was very easy to stretch the SPF in a non-visual situation. We cut the fiber connective tissue when meeting resistance. The posterior layer of the SPF was dense and difficult to penetrate. We used the other hand to feel the position of the scissor’ tip to ensure the scissor was in the correct layer. The time of establishing the OSC was only 3–4 min, much less than any traditional methods. The overall operation time is greatly shortened. Therefore, the technique is minimally invasive.

As shown in our results, compared with the COA, the operating time of BAA was longer. It was not hard to understand for it was easier to operate in an open incision. However, we thought it was worthy for aesthetic result at a cost of about more 30 min per operation. And we thought we could shorten the operation time with the improvement of skill and accumulations of experiences. The number of lymph nodes dissected, postoperative hospitalization time and incidence of postoperative complications did not show significant differences between the two groups. Lee et al. (13) reported that the average number of retrieved lymph nodes was 3.63 ± 2.1 in endoscopic thyroidectomy and 3.82 ± 3.28 in COA. Considering unilateral central lymph node dissection, it was not too small compared with the number of recent studies. The results showed the BAA technique was safe and reliable. The less intraoperative bleeding in BAA also showed the superiority of this technique.

Furthermore, The BAA group exhibits lower pain scores than COA group. Some researchers explained it was because the BAA just exploit the subcutaneous tunnels and didn't need cutting off the neck platysma (14). The skin and subcutaneous tissue were well combined when the trocar was extracted. However, COA need cut off the neck platysma to expose the thyroid bed. In addition, we added lidocaine and ropivacaine in the local swelling anesthetic solution, which also played a great role in reducing the pain of the patient.

In our procedure, we adopted capsule dissection technique, and the RLN and parathyroid gland were magnified on a videoscope. Therefore, their preservation was easy. There was one patient whose symptom of transient vocal cord palsy spontaneously disappeared after 2 months in the BAA group. This might be caused by the heat conduction damage of the energy device during the operation. This situation did not happen again after we cooled the ultrasonic scalpel before the next step carefully.

Of note, patients in the BAA group had significantly higher satisfaction of cosmetic scores with shorter incision length compared with patients in the COA group. As we described, BAA thyroidectomy is a highly feasible and safe surgical procedure, special for the younger female patients (15).

The present study provides robust evidence supporting the clinical superiority of the BAA over the COA in carefully selected patients, particularly young women with high cosmetic expectations. Even after rigorous propensity score full matching that improved balance for distance (SMD from 3.04 to 0.65) and age (1.69–0.27) but worsened balance for BMI (0.99–1.62), tumor size (0.33 to −0.56), and multiplicity (0.05 to −0.56), BAA retained highly significant advantages in the most patient-centered outcomes: an adjusted 1.3-point higher cosmetic satisfaction score (95% CI 0.98–1.64, *p* = 7.93 × 10⁻^1^⁵), approximately 15 mL less intraoperative blood loss (*p* = 1.22 × 10⁻⁹), and lower postoperative pain scores. The consistency of these findings across both unadjusted and PSM-adjusted analyses strongly suggests that the benefits of BAA are not merely attributable to younger age or other measured confounders.

To our knowledge, this is one of the first studies in endoscopic thyroidectomy to apply DCA. Despite the modest sample within the BAA group, the full prediction model (age + BMI + tumor size + unilateral procedure) demonstrated clear positive net benefit over an age-only strategy across a wide range of clinically reasonable risk thresholds, translating into correctly identifying an additional 30–35 truly highly satisfied patients per 1,000 without increasing unnecessary interventions. This exploratory but promising result highlights the feasibility of using simple preoperative clinical variables to further personalize patient selection and counseling within the BAA-eligible population.

Thus, the combined use of advanced propensity score methodology and DCA (not commonly reported in the endoscopic thyroidectomy literature) represents a significant methodological strength. These approaches enhance the credibility and real-world relevance of our findings, reinforcing that BAA offers substantial cosmetic and perioperative benefits for appropriately selected patients with papillary TC.

Several important limitations must be acknowledged with respect to the PSM analyses. First, although full matching with a logistic regression propensity score (full + glm) achieved the greatest reduction in SMD among the six methods evaluated, substantial residual imbalance persisted for key covariates—notably BMI (post-matching SMD=1.62) and tumor size (SMD=−0.56). Conventional thresholds for acceptable balance are typically require |SMD| < 0.1–0.25; values exceeding 1.0 indicate severe remaining confounding. Consequently, even after PSM, estimates of treatment effect (particularly for outcomes potentially influenced by body habitus or lesion size) may still be biased.

Second, the interpretation of certain PSM results warrants caution. In the unmatched analysis, the number of retrieved central lymph nodes showed no significant difference between groups (3.1 ± 0.7 vs. 3.2 ± 0.5, *p* = 0.322), yet after full matching the COA was associated with retrieval of 0.37 more nodes (*p* = 0.0068). This reversal from non-significant to significant likely reflects changes in the effective sample composition and residual confounding rather than a true oncologic superiority of COA, highlighting the instability of effect estimates when balance remains imperfect.

Most critically, patient allocation to BAA vs. COA was driven primarily by patients’ aesthetic demand and preoperative cosmetic expectations, which constitute a strong, patient-driven, and largely unmeasured confounder that may directly influence subjective outcomes such as cosmetic satisfaction and pain scores. Younger patients (mean age 29.0 years in BAA vs. 43.5 years in COA) who actively requested a scarless visible scar were preferentially offered BAA. Propensity score methods can only adjust for observed and measured covariates; they cannot balance unmeasured factors such as strength of cosmetic expectation, psychological tolerance of neck scars, or willingness to accept longer operative time for better appearance. These unmeasured factors are likely to directly influence patient-reported outcomes (pain and especially cosmetic satisfaction scores. Therefore, even with apparently improved (though still incomplete) covariate balance after PSM, systematic differences in patient expectations and subjective perception probably persist, limiting the ability of PSM to produce unbiased estimates of the true treatment effect on these subjective outcomes. The highly significant post-matching differences in cosmetic satisfaction (≈1.3 points lower in COA) and pain scores should thus be interpreted as upper-bound estimates of the approaches’ true causal effects.

Another important limitation pertains to the scope and interpretation of the DCA. The primary aim of this study was to compare cosmetic and clinical outcomes between the BAA and COA. In the COA group, the visible anterior neck scar (typically 6–10 cm) makes it virtually impossible for patients to achieve the same level of “extremely high” cosmetic satisfaction (score >9 out of 10) that is attainable with BAA. Consequently, the binary outcome of “high cosmetic satisfaction” used in the prediction model is almost exclusively realized in the BAA group and is not meaningfully comparable across the two surgical approaches.

Therefore, constructing a single prediction model and performing DCA on the entire mixed cohort (BAA + COA) would not be clinically sensible, as it would force an outcome that is structurally unattainable in the COA arm. The current DCA was thus intentionally restricted to the 42 patients who underwent BAA, with the more modest but realistic goal of identifying, among patients already selected for BAA (predominantly young females with strong cosmetic demands), which preoperative characteristics are associated with the highest likelihood of achieving excellent cosmetic satisfaction. This approach provides supplementary information for preoperative counseling and patient selection within the BAA-eligible population rather than directly informing the binary choice between BAA and COA.

We fully acknowledge that this design limits the ability of the DCA to serve as a decision-support tool for choosing between the two surgical approaches, which would have been the ideal application in a comparative study. The present analysis should therefore be interpreted as an exploratory evaluation of potential predictors of optimal cosmetic outcome specifically after BAA, rather than as definitive evidence for surgical decision-making between BAA and COA. Additionally, the small sample size of only 42 BAA patients remains a critical limitation, rendering the net benefit curve unstable and prone to overfitting. Future studies with substantially larger BAA cohorts are needed before any prediction model can be considered reliable for routine clinical use.

## Conclusion

In summary, compared with the COA, BAA thyroidectomy is more desirable in terms of minimum invasiveness and cosmetic outcomes. It is very essential to establish the operating space rapidly and simply. We believe that with the accumulation of surgical experience and continuous improvement of endoscopic instruments, the BAA thyroidectomy will be widely used and can be performed for the treatment of more complex thyroid diseases in the future. However, a large-scale, prospective, multicenter clinical study should be conducted to validate the superiority of the BAA.

## Data Availability

The original contributions presented in the study are included in the article/Supplementary Material, further inquiries can be directed to the corresponding author.
